# LncRNA DLGAP1‐AS1 accelerates glioblastoma cell proliferation through targeting miR‐515‐5p/ROCK1/NFE2L1 axis and activating Wnt signaling pathway

**DOI:** 10.1002/brb3.2321

**Published:** 2021-09-18

**Authors:** Zixuan Wang, Yipeng Han, Qifeng Li, Baocheng Wang, Jie Ma

**Affiliations:** ^1^ Department of Pediatric Neurosurgery Xinhua Hospital Affiliated to Shanghai Jiaotong University School of Medicine Shanghai China

**Keywords:** DLGAP1‐AS1, glioblastoma, miR‐515‐5p, NFE2L1, ROCK1

## Abstract

**Introduction:**

Glioblastoma (GBM), the primary malignant tumor in the central nervous system, features high aggressiveness and mortality. Long noncoding RNAs (lncRNAs) can exert the crucial function in regulating various human diseases, including GBM. However, the function and mechanism of lncRNA DLGAP1 antisense RNA 1 (DLGAP1‐AS1) in GBM remain still unknown.

**Methods:**

DLGAP1‐AS1 expression in GBM cells was detected by RT‐qPCR. Functional assays were conducted to determine GBM cell proliferation and apoptosis. RIP, RNA pull down, and luciferase reporter assay were applied for measuring the interplay of DLGAP1‐AS1 with other RNAs.

**Results:**

DLGAP1‐AS1 was distinctly upregulated in GBM cells. DLGAP1‐AS1 depletion inhibited cell proliferation, but induced apoptosis. MiR‐515‐5p could be sponged by DLGAP1‐AS1 in GBM cells and to repress cell proliferation in GBM. Further, Rho‐associated coiled‐coil containing protein kinase 1 (ROCK1) and Nuclear factor erythroid‐2 like 1 (NFE2L1) were confirmed as the target gene of miR‐515‐5p. Wnt signaling pathway could be activated by DLGAP1‐AS1 via regulating ROCK1 and NFE2L1 expression. Rescue assays proved that overexpression of both ROCK1 and NFE2L1 could totally reverse the inhibitory effect of silencing DLGAP1‐AS1 on GBM cell proliferation.

**Conclusion:**

LncRNA DLGAP1‐AS1 accelerated cell proliferation in GBM via targeting miR‐515‐5p/ROCK1/NFE2L1 axis and activating Wnt signaling pathway.

## Graphical abstract

1. DLGAP1‐AS1 is significantly upregulated in GBM cells and facilitates cell proliferation.

3. NFE2L1 and ROCK1 are targeted by miR‐515‐5p in GBM.

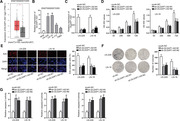

## 1 INTRODUCTION

Glioblastoma (GBM) is the primary malignant tumor in the central nervous system (Wirsching et al., 2016). It is extremely aggressive, and the disease develops very fast (Wirsching et al., 2016). At present, the main treatment strategies of GBM include surgical operation, chemotherapy and radiotherapy (Fontaine & Paquis, 2010). However, due to its aggressive and recurrent nature, the overall prognosis of patients is very poor. Even more depressing is that, so far, the median overall survival of patients with GBM is only about 14 months (Fontaine & Paquis, 2010; Wang et al., 2013). Recently, molecular targeting therapy has become the research highlights in many human cancers, including GBM (Davis, 2016). Nevertheless, GBM pathogenesis is not elucidated. Thus, it is urgent to develop new treatment methods for curing GBM.

Long noncoding RNAs (lncRNAs) are the RNA transcripts comprising over 200 nucleotides in length without the capability of coding proteins (Jathar et al., 2017). An increasing number of studies have indicated the fact that lncRNAs are biologically functional molecules, which are in sharp contrast to the original statement that lncRNAs are transcriptional noise (Kashi et al., 2016). Importantly, accumulating evidences have confirmed that the dysregulation of lncRNAs is closely associated with human cancer progression, and can be involved in the regulation of cancer development (Bhan, Soleimani & Mandal, 2017). For instance, lncRNA PTCSC3 was reported as a biomarker for the treatment and prognosis of gastric cancer (Zhang et al., 2020). LncRNA FTH1P3 was highly expressed in cervical cancer cells and it could facilitate cell proliferation via interacting with miR‑145 (Lv & Zhang, 2020). LncRNA MATN1‐AS1 was downregulated in GBM cells and could repress cell proliferation and invasion through regulating RELA (Han et al., 2019). And high expression of lncRNA GAPLINC was proved to accelerate metastasis of GBM through sponging miR‐331‐3p (Chen et al., 2019).

LncRNA DLGAP1 antisense RNA 1 (DLGAP1‐AS1) has been identified in several cancers to exert the regulatory functions. For example, DLGAP1‐AS1 could accelerate tumorigenesis and EMT process of hepatocellular carcinoma through regulating miR‐26a/b‐5p/IL‐6/JAK2/STAT3 axis and Wnt/β‐catenin pathway (Lin et al., 2020). Further, DLGAP1‐AS1 could facilitate the cell invasion of gastric cancer by interacting with miR‐628‐5p and upregulating AEG‐1 (Deng et al., 2020). However, its detained function and mechanism in GBM are still unclear.

A flow of researches has indicated that lncRNA can interact with microRNAs (miRNAs) in cancer cells. MiRNA is the small endogenous RNA and it can negatively regulate their target messenger RNAs (mRNAs) at post transcription level (Lu & Rothenberg, 2018). The biological role of miRNAs in the regulation of cell processes has been discovered earlier and miRNAs also take part in assorted cancer progression (Mishra et al., 2016). For example, miR‐758‐5p repressed cell proliferation in GBM through targeting ZBTB20 (Liu et al., 2018). HMGB3 facilitated metastasis of GBM and it was negatively regulated by miR‐200b‐3p and miR‐200c‐3p (Liu et al., 2018). MiR‐340‐5p could also inhibit aggressiveness of GBM through targeting Bcl‐w and Sox2 (Kim et al., 2019). However, the function of miR‐515‐5p in GBM and its interaction with DLGAP1‐AS1 are still unclear.

In our research, we aimed to explore the detailed function and mechanism of DLGAP1‐AS1 in GBM cells. Further, the interplay of DLGAP1‐AS1 and other RNAs was also investigated.

## 2 MATERIALS AND METHODS

### 2.1 Cell culture

Four human GBM cell lines (LN‐229, LN‐18, A‐172and T98G) were obtained from ATCC (Manassas, VA, USA) and the normal human astrocytes (NHAs) were obtained from YAJI Biotechnology Co.,Ltd. (Shanghai, China). Above cell lines were all cultured in Dulbecco's Modified Eagle Medium (Gibco, Grand Island, NY, USA) supplied with 10% fetal bovine serum (FBS; Gibco). The mediums were cultivated with 5% CO_2_ at 37 °C.

### 2.2 Cell transfection

For silencing the expression of DLGAP1‐AS1, the shRNAs specific targeting to DLGAP1‐AS1 (sh‐DLGAP1‐AS1) and its negative control (sh‐NC) were purchased from GenePharma (Shanghai, China). Then, pcDNA3.1/ROCK1, pcDNA3.1/NFE2L1 and their NC were also obtained from GenePharma for upregulating their expression. The miR‐515‐5p mimics and NC mimics were obtained from Ribobio (Guangzhou, China). Cell transfection was implemented with the utilization of Lipofectamine 3000 (Invitrogen) for 48 hours.

### 2.3 RT‐qPCR analysis

The extraction of total RNA was obtained by TRIzol reagent (Invitrogen, Carlsbad, CA, USA). Then the reverse transcribing RNA into cDNA was conducted by Reverse Transcription Kit (A5001, Promega, China). Next, qPCR was performed with SYBR Green Mix (Vazyme Biotech, Nanjing, China). 2^−ΔΔCt^ was utilized to count the relative gene expression. GAPDH or U6 acted as control.

### 2.4 CCK‐8 assay

After transfection, cells were plated into the 48‐well plates at a density of 1 × 10^3^ cells per well. Cells were cultivated for 0, 24, 48, 72 hours with CCK‐8 reagent (Dojindo, Kumamoto, Japan) and cultured for 4 hours. The optical density (OD) value was analyzed on by a microplate reader.

### 2.5 EdU assay

EdU assay was conducted with EdU kit (RiboBio, Guangzhou, China) in line with the protocols of supplier. We seeded cells in 96‐well plates and then they were subjected to EdU staining. Nuclei were stained with DAPI. Finally, the fluorescence microscope (Olympus, Tokyo, Japan) was applied for observation. Biorepeats were run in triplicate.

### 2.6 Colony formation

Clonogenic cells of LN‐229 and LN‐18 were put in the 6‐well plates. After 14 days of cultivation, cells were subjected to fixation with methanol and then they were dyed with 0.5% crystal violet. Finally, the number of colonies was calculated manually.

### 2.7 Caspase‐3/8/9 activity assay

The activity of caspase‐3/8/9 was separately detected in LN‐229 and LN‐18 cells employing Caspase‐3/8/9 assay kit (Abcam, Cambridge, MA, USA) in line with user guides. After 72 hours of cultivation, cells in 6‐well plates were detected through microplate reader at 405 nm.

### 2.8 Flow cytometry analysis

Cell apoptosis capability was evaluated by utilizing the Annexin V‐FITC apoptosis kit according to supplier's instruction. Specifically, 2 × 10^5^ transfected cells were collected and then rinsed by PBS twice, and then stained by Annexin V‐FITC for 10 minutes in dark room. Flow cytometry was applied for observation.

### 2.9 Western blot

First, we obtained the total cell protein samples and then isolated them by electrophoresis with 10% SDS‐PAGE, followed by transferring to the PVDF membranes. Then the membranes were blockaded with 5% skimmed dried milk. Next, they were cultivated with the primary antibodies (Abcam, Cambridge, MA, USA) at 4 °C for one night. After that, the membranes were incubated with a HRP‐conjugated secondary antibody for 1 hour. In the end, the band intensity was analyzed by enhanced chemiluminescence reagent (Santa Cruz Biotechnology, Santa Cruz, CA, USA).

### 2.10 Subcellular fractionation assay

The isolation of cytoplasmic and nuclear RNA of 1 × 10^6^ GBM cells was conducted with PARIS™ Kit (Invitrogen) in line with the protocols of supplier. After purification, the isolated RNAs were analyzed by RT‐qPCR. GAPDH and U6 served as control.

### 2.11 RNA pull down

Biotin‐labeled wild or mutant of miR‐515‐5p and biotin‐labeled DLGAP1‐AS1 were obtained from Pierce™ Magnetic RNA–protein pull‐down kit (Thermo fisher, IL, USA). After obtaining the cell lysates in RIPA buffer, we mixed them with the biotinylated RNA and magnetic beads. After centrifuging, relative enrichment of RNAs was analyzed by RT‐qPCR.

### 2.12 RIP analysis

On the basis of protocols of supplier, Magna RIP™ RNA‐Binding Protein Immunoprecipitation Kit (Millipore, Billerica, MA, USA) was applied for implementing this assay. The LN‐229 and LN‐18 cell lysates obtained from RIP lysis buffer were employed for immunoprecipitation with magnetic beads and Anti‐AGO2 and Anti‐IgG (Millipore, Billerica, MA, USA). After rinsing, the precipitated RNA was analyzed by RT‐qPCR.

### 2.13 Luciferase reporter assay

The wild type (WT) and mutated (MUT) miR‐515‐5p binding sites to DLGAP1‐AS1 or ROCK1 or NFE2L1 were obtained for constructing pmirGLO‐DLGAP1‐AS1‐WT/MUT and pmirGLO‐ROCK1‐WT/MUT or NFE2L1‐WT/MUT. Then they were subjected to cotransfection with indicated plasmids into cells for 48 hours. Following, the luciferase activity was analyzed via Luciferase Reporter Assay System (Promega, Madison, WI, USA). As to the detection of Wnt/β‐catenin pathway activity, the reporter vectors TOP/FOP‐Flash (Addgene, Cambridge, MA, USA) were cotransfected to cells with sh‐DLGAP1‐AS1 or sh‐NC.

### 2.14 Statistical analysis

Each assay in our research was performed at least three times. Mean ± SD was used to represent the data. Statistical analysis was conducted with SPSS 23.0. Group difference was analyzed by the Student's *t*‐test or ANOVA. *p* < .05 was considered as statistically significance.

## 3 RESULTS

### 3.1 DLGAP1‐AS1 displays aberrantly high expression in GBM and induces cell malignant behaviors

**FIGURE 1:**
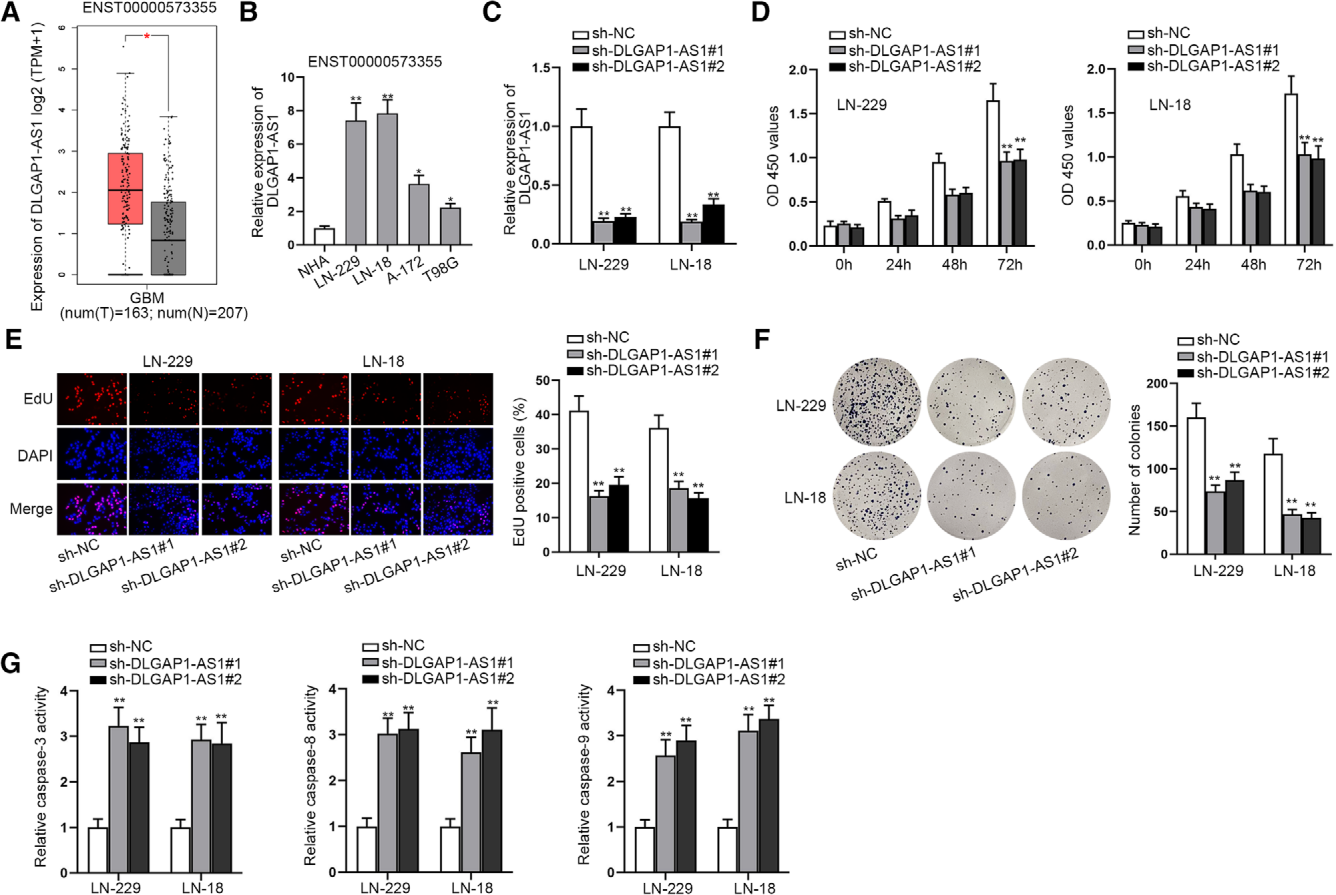
DLGAP1‐AS1 is distinctly overexpressed in GBM cells and facilitates cell proliferation. (a) DLGAP1‐AS1 (ENST00000573355) expression in GBM tissues was obtained from GEPIA. (b) DLGAP1‐AS1 (ENST00000573355) expression in GBM cell lines and NHAs was detected via RT‐qPCR. (c) DLGAP1‐AS1 interference efficiency in cells was determined by means of RT‐qPCR analysis. (d) CCK‐8 assays were applied for measuring cell viability in sh‐DLGAP1‐AS1‐transfected cells and sh‐NC‐transfected cells. (e–f) EdU and colony formation assays were utilized for the assessment of cell proliferation when DLGAP1‐AS1 was silenced in cells. (g) Caspase‐3/8/9 activity assay was employed for detecting cell apoptosis after DLGAP1‐AS1 was knocked down in cells. ^*^ *p* < .05, ^**^ *p* < .01

Dysregulation of lncRNAs often occurred in cancer cells. Therefore, we determined DLGAP1‐AS1 expression in GBM tissues and cells first. Since DLGAP1‐AS1 has six transcripts, we searched on online database GEPIA (http://gepia.cancer‐pku.cn/) and found that DLGAP1‐AS1‐005 (ENST00000573355) and DLGAP1‐AS1‐002 (ENST00000576606) were expressed higher in GBM tissues than that in normal brain tissues (Figures 1a and S1a). Moreover, we applied RT‐qPCR to measure the expression level of these two transcripts in GBM cell lines (LN‐229, LN‐18, A‐172, and T98G) and the normal human astrocytes (NHAs). RT‐qPCR measurement also confirmed DLGAP1‐AS1‐005 (ENST00000573355) but not DLGAP1‐AS1‐002 (ENST00000576606) DLGAP1‐AS1 was expressed higher in GBM cell lines than that in NHAs (Figures 1b and S1b). Therefore, we chose DLGAP1‐AS1‐005 (ENST00000573355, named as DLGAP1‐AS1) in our subsequent study. Especially, DLGAP1‐AS1 expression in LN‐229 and LN‐18 cells was higher than that in other GBM cells. Thus, we selected these two cells for the further assays. Considering the high expression of DLGAP1‐AS1, we decided to transfect the specific shRNA against DLGAP1‐AS1 into LN‐229 and LN‐18 cells to knock down the expression of DLGAP1‐AS1. Then, RT‐qPCR results displayed an effective interference efficiency of sh‐DLGAP1‐AS1 (Figure 1c). After that, we conducted a chain of loss‐of‐functional assays. Through CCK‐8 assay, we discovered that the optical density value of sh‐DLGAP1‐AS1‐transfected cells was evidently lower than that of negative control cells, suggesting cell viability could be damaged by silencing DLGAP1‐AS1 (Figure 1d). Then EdU assay was performed for detection of cell proliferation and the results displayed that the ratio of EdU positive cells was notably decreased in cells transfected with sh‐DLGAP1‐AS1, which indicated the repression of cell proliferation by DLGAP1‐AS1 knockdown (Figure 1e). Further, colony formation assay also proved cell proliferation could be suppressed by DLGAP1‐AS1 downregulation since the number of colonies was reduced in the sh‐DLGAP1‐AS1 transfection groups (Figure 1f). However, cell apoptosis was enhanced by DLGAP1‐AS1 depletion. In caspase‐3/8/9 activity assays, we found that the relative activity of caspase‐3/8/9 was significantly elevated in cells transfected with sh‐DLGAP1‐AS1 (Figure 1g). Also, in flow cytometry assay, cell apoptosis rate was increased by DLGAP1‐AS1 knockdown (Figure S2a). Moreover, it was obtained from GEPIA that the overall survival of GBM patients with high DLGAP1‐AS1 was poorer than those with low DLGAP1‐AS1 (Figure S2b). In short, DLGAP1‐AS1 facilitated proliferation but inhibited apoptosis of GBM cells.

### 3.2 DLGAP1‐AS1 binds to miR‐515‐5p by acting as a ceRNA in GBM cells

**FIGURE 2:**
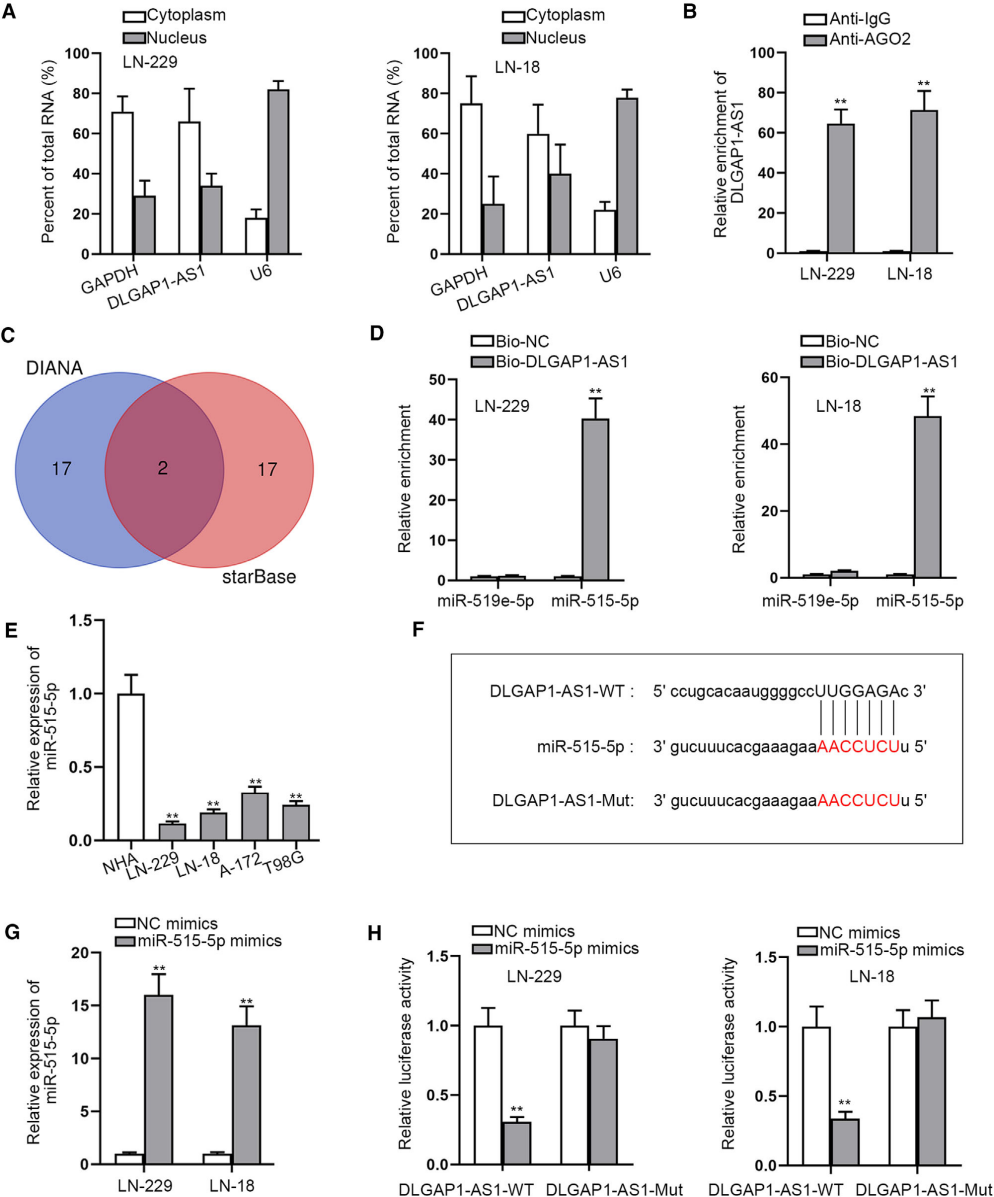
DLGAP1‐AS1 binds to miR‐515‐5p as a ceRNA in GBM cells. (a) Subcellular fractionation assay was done for detecting the distribution of DLGAP1‐AS1 in LN‐229 and LN‐18 cells. (b) RIP assay detected the combination between DLGAP1‐AS1 and AGO2 protein. (c) DIANA database and starBase database predicted two candidate miRNAs that might bind with DLGAP1‐AS1. (d) The more suitable miRNA for DLGAP1‐AS1 was determined after RNA pull‐down assay. (e) MiR‐515‐5p expression in GBM cells was detected via RT‐qPCR. (f) Binding site between DLGAP1‐AS1 and miR‐515‐5p was predicted by starBase. (g) The overexpression efficiency of miR‐515‐5p mimics was detected via RT‐qPCR. (h) Interaction between DLGAP1‐AS1 and miR‐515‐5p was observed through luciferase reporter assay. ^**^ *p* < .01

LncRNAs are confirmed to take part in cancer progression through interacting with miRNAs (Paraskevopoulou & Hatzigeorgiou, 2016). Competing endogenous RNA (ceRNA) network is a popular regulatory mechanism of RNA interaction and lncRNA can act as a ceRNA to sponge miRNA for releasing the miRNA inhibition on mRNA expression at posttranscriptional level (Smillie et al., 2018). For exploring the ceRNA role of DLGAP1‐AS1, we first detected its distribution in GBM cells. Through subcellular fractionation assay, we discovered that DLGAP1‐AS1 was mainly located in cytoplasm of LN‐229 and LN‐18 cells (Figure 2a), suggesting DLGAP1‐AS1 regulated at posttranscriptional level. Furthermore, RIP assay also indicated that DLGAP1‐AS1 abundantly enriched in Anti‐AGO2 groups, indicating DLGAP1‐AS1 existed in AGO2 antibody‐associated complex (Figure 2b). These results implied that DLGAP1‐AS1 could interact with the specific miRNA in GBM cells. Thus, we utilized starBase database (http://starbase.sysu.edu.cn/index.php) and DIANA database (http://carolina.imis.athena‐innovation.gr/diana_tools/web/index.php) to predict the possible miRNAs that could bind with DLGAP1‐AS1. After prediction, we obtained two candidate miRNAs (miR‐519e‐5p and miR‐515‐5p) (Figure 2c). To select out the more suitable miRNA, we performed RNA pull‐down assay. In accordance with the results, we discovered that only miR‐515‐5p could be substantially pulled down by biotinylated DLGAP1‐AS1 probe in cells, while miR‐519e‐5p could not be pulled down (Figure 2d). It proved that miR‐515‐5p could bind with DLGAP1‐AS1. In the following RT‐qPCR assay, we observed the notable downregulation of miR‐515‐5p in GBM cells compared with the NHAs (Figure 2e). After that, for further validating the binding between DLGAP1‐AS1 and miR‐515‐5p, we predicted their binding sites through starBase database, which were displayed in Figure 2f. After overexpressing miR‐515‐5p in cells by transfecting miR‐515‐5p mimics (Figure 2g), we performed luciferase reporter assays. The DLGAP1‐AS1 sequence including the putative or mutated miR‐515‐5p binding sites was subcloned to the luciferase reporter vector, creating DLGAP1‐AS1‐WT or DLGAP1‐AS1‐Mut luciferase reporter plasmids. With miR‐515‐5p mimics cotransfection, the luciferase activity of DLGAP1‐AS1‐WT was hampered, while that of DLGAP1‐AS1‐Mut showed no change (Figure 2h). In short, these results proved that DLGAP1‐AS1 bound to miR‐515‐5p in GBM cells as a ceRNA.

### 3.3 MiR‐515‐5p overexpression represses malignant behaviors of GBM cells

**FIGURE 3:**
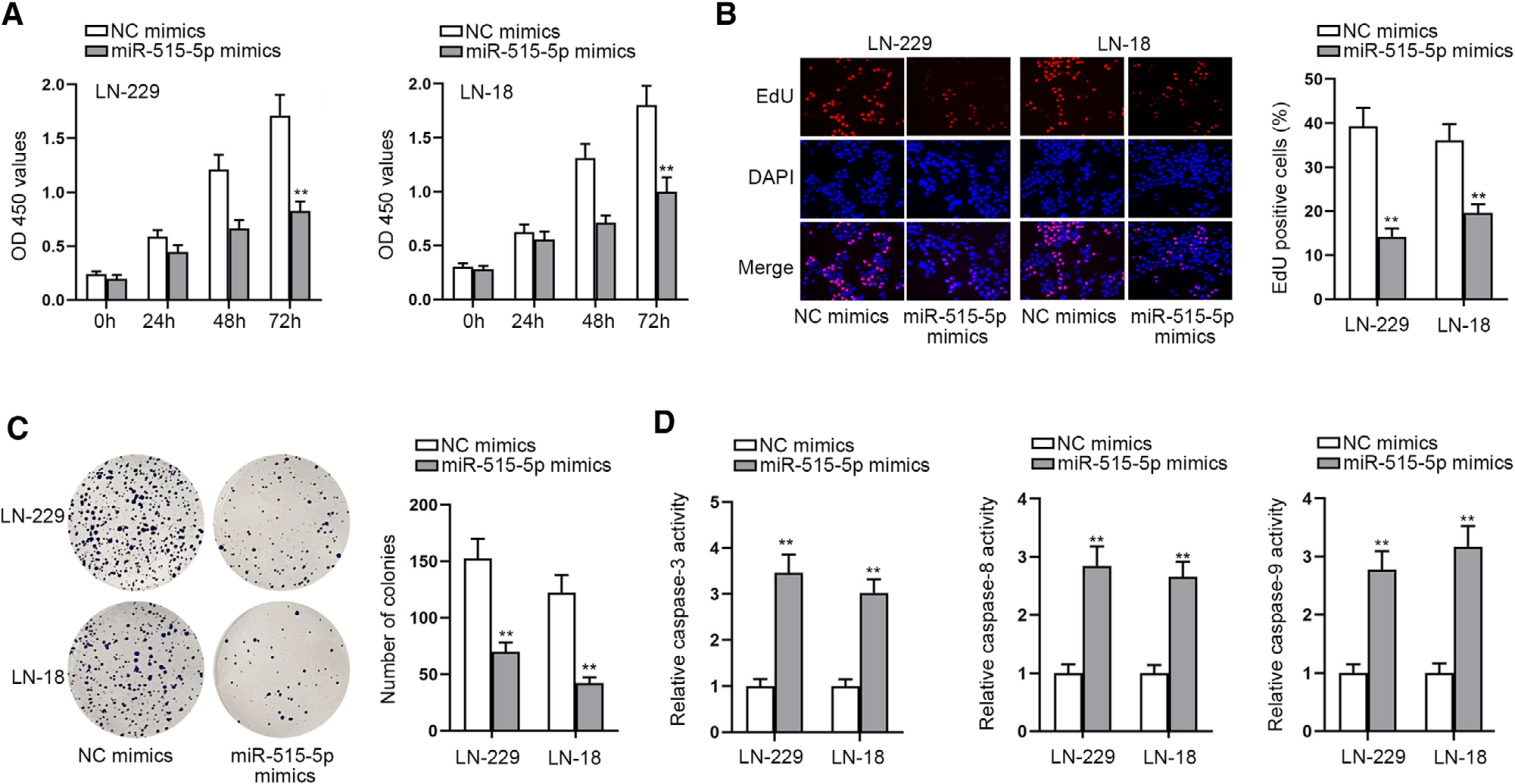
MiR‐515‐5p overexpression represses cell proliferation in GBM. (a) CCK‐8 assay was done for the evaluation of cell viability in response to the transfection of different plasmids including miR‐515‐5p mimics and NC mimics. (b–c) EdU and colony formation assays were applied for the investigation into cell proliferation when miR‐515‐5p was overexpressed in cells. (d) Caspase‐3/8/9 activity assay was employed for detecting cell apoptosis under the influence of miR‐515‐5p upregulation. ^**^ *p* < .01

The biological function of miR‐515‐5p in GBM cells was detected via a series of functional assays. CCK‐8 assay results verified that cell viability in miR‐515‐5p mimics‐transfection groups was remarkably reduced in comparison with negative control groups (Figure 3a). The subsequent EdU and colony formation assays further proved that cell proliferation was impeded by miR‐515‐5p upregulation since the number of EdU positive cells and the number of colonies declined due to the transfection of miR‐515‐5p mimics (Figure 3b‐c). Finally, it was indicated through caspase‐3/8/9 activity detection that the relative caspase‐3/8/9 activity was enhanced in cells transfected with miR‐515‐5p mimics, and flow cytometry assay validated cell apoptosis rate was elevated by miR‐515‐5p overexpression, both indicating cell apoptosis was promoted (Figures 3d and S2c). In a word, miR‐515‐5p overexpression hindered cell proliferation and accelerated cell apoptosis in GBM.

### 3.4 NFE2L1 and ROCK1 are targeted by miR‐515‐5p in GBM cells

**FIGURE 4:**
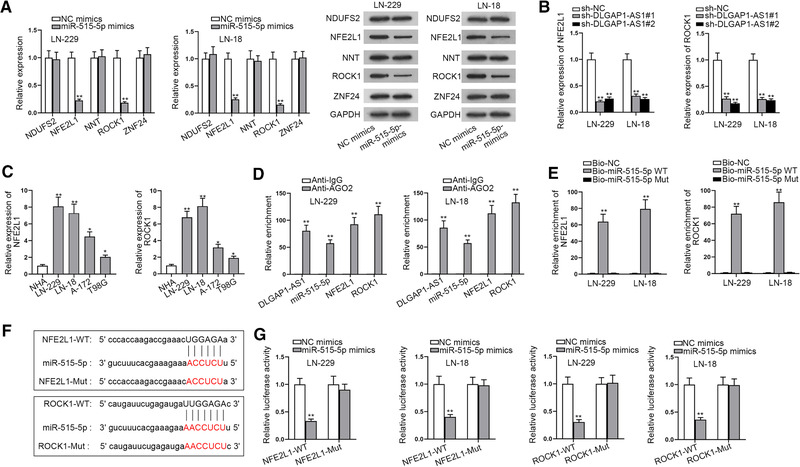
NFE2L1 and ROCK1 are targeted by miR‐515‐5p in GBM cells. (a) RT‐qPCR analyzed the expression of candidate mRNAs and western blot quantified the relevant protein expression in cells transfected with miR‐515‐5p mimics. (b) RT‐qPCR was applied for analyzing the expression of NFE2L1 and ROCK1 in cells transfected with sh‐DLGAP1‐AS1. (c) NFE2L1 and ROCK1 expression in GBM cell lines and NHAs was measured through RT‐qPCR. (d) RIP assay was utilized to prove whether DLGAP1‐AS1, miR‐515‐5p, NFE2L1, and ROCK1 coexisted in RISC complex. (e) RNA pull‐down assay confirmed the combination of miR‐515‐5p and NFE2L1 or ROCK1. (f) The binding site between miR‐515‐5p and NFE2L1 or ROCK1 was predicted from starBase. (g) Luciferase reporter assays were applied for validating the binding sites. ^*^ *p* < .05, ^**^ *p* < .01

Subsequently, the downstream target mRNAs of miR‐515‐5p was further explored. Through searching the starBase database with the specific conditions (CLIP‐Data ≥ 5; Degradome‐Data ≥ 3; pan‐Cancer ≥ 6), we found five mRNAs that might bind with miR‐515‐5p. We first transfected cells with miR‐515‐5p mimics and tested the change in expression of these mRNAs through RT‐qPCR. We found that only expression levels of Nuclear factor erythroid‐2 like 1 (NFE2L1) and Rho associated coiled‐coil containing protein kinase 1 (ROCK1) were reduced by miR‐515‐5p overexpression (Figure 4a). Thus, we further evaluated the expression of the two mRNAs in cells transfected with sh‐DLGAP1‐AS1, and we found that level of both mRNAs was decreased by DLGAP1‐AS1 depletion (Figure 4b). It indicated that NFE2L1 and ROCK1 could inversely associate with miR‐515‐5p but positively associate with DLGAP1‐AS1. Then we separately detected NFE2L1 and ROCK1 expression in GBM cell lines and NHAs. RT‐qPCR analysis displayed that both of them were highly expressed in GBM cells compared with NHAs (Figure 4c). Therefore, we speculated that miR‐515‐5p may target NFE2L1 and ROCK1 simultaneously in GBM cells. Following, RIP assays were carried out in GBM cell extracts utilizing the AGO2 antibody. The results demonstrated that all of the four RNAs (DLGAP1‐AS1, miR‐502‐3p, NFE2L1, and ROCK1) coexisted in RISC complex, suggesting the ceRNA network may exist in GBM cells (Figure 4d). The further RNA pull‐down assay proved that NFE2L1 and ROCK1 could be notably pulled down by the biotinylated miR‐515‐5p WT probe, which indicated that both NFE2L1 and ROCK1 could bind with miR‐515‐5p (Figure 4e). For the sake of validating their combination, the putative binding sites between NFE2L1/ROCK1 and miR‐515‐5p were obtained (Figure 4f) and luciferase reporter assays were performed. The results illustrated that upregulation of miR‐515‐5p reduced the luciferase activity of NFE2L1‐WT and ROCK1‐WT, which further confirmed the binding relationship between miR‐515‐5p and NFE2L1 or ROCK1 (Figure 4g). Overall, NFE2L1 and ROCK1 were targeted by miR‐515‐5p in GBM cells.

### 3.5 DLGAP1‐AS1 activates Wnt signaling pathway via regulating NFE2L1 and ROCK1

**FIGURE 5:**
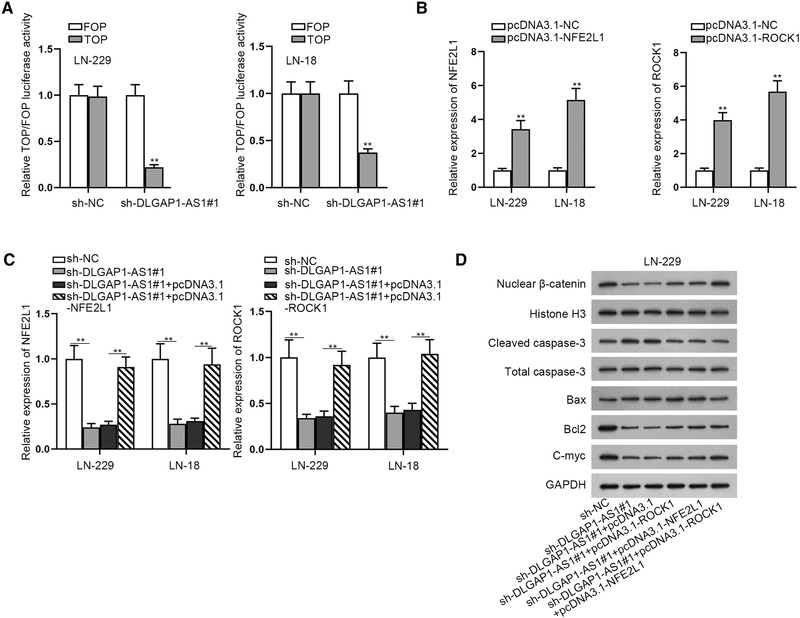
DLGAP1‐AS1 activates Wnt signaling pathway via regulating NFE2L1 and ROCK1. (a) TOP/FOP assays were applied for detecting the activity of Wnt signaling pathway. (b) The overexpression efficiency of pcDNA3.1‐NFE2L1 or pcDNA3.1‐ROCK1 was determined based on RT‐qPCR analysis. (c) NFE2L1 or ROCK1 expression was detected in cells after different groups of transfections, specifically, sh‐NC, sh‐DLGAP1‐AS1#1 and sh‐DLGAP1‐AS1#1+pcDNA3.1‐NFE2L1 or sh‐DLGAP1‐AS1#1+pcDNA3.1‐ROCK1. (d) Western blot assays were utilized for evaluating the influence of DLGAP1‐AS1, NFE2L1, and ROCK1 on the level of proteins related to Wnt signaling pathway. ^**^ *p* < .01

It was reported that ROCK1 and NFE2L1 could activate the activity of Wnt signaling pathway in human cancers. ROCK1 has been confirmed as the main gene of the Wnt signaling pathway (Mohammadi‐Yeganeh et al., 2019; Xie et al., 2020). The recent research also reported that NFE2L1 could regulate lung adenocarcinoma progression through Wnt pathway (Wei et al., 2020). Thus, we investigated whether DLGAP1‐AS1 could activate Wnt signaling pathway via regulating NFE2L1 or ROCK1 in GBM cells. First, through luciferase activity assays, we found that the relative TOP/FOP luciferase activity was repressed in cells transfected with sh‐DLGAP1‐AS1, indicating that Wnt signaling pathway was inactivated by DLGAP1‐AS1 depletion (Figure 5a). Then we separately overexpressed NFE2L1 or ROCK1 in GBM cells by transfecting pcDNA3.1‐NFE2L1 or pcDNA3.1‐ROCK1. RT‐qPCR assay displayed that their expression was effectively enhanced by the two overexpression vectors (Figure 5b). Following, we further discovered that NFE2L1 expression repressed by sh‐DLGAP1‐AS1 could be rescued by pcDNA3.1‐NFE2L1 cotransfection. Also, ROCK1 expression inhibited by sh‐DLGAP1‐AS1 could be countered by addition of pcDNA3.1‐ROCK1 (Figure 5c). Next, we implemented western blot assays to detect the level change of proteins related with Wnt signaling pathway, which manifested that the protein level of nuclear β‐catenin was repressed after silencing DLGAP1‐AS1, which was partly recovered by cotransfection of pcDNA3.1‐ROCK1. Similarly, the inhibited nuclear β‐catenin level caused by DLGAP1‐AS1 depletion also could be partly rescued by cotransfection of pcDNA3.1‐NFE2L1. Further, we discovered that cotransfection of pcDNA3.1‐ROCK1 and pcDNA3.1‐NFE2L1 could totally recover the reduced protein level of nuclear β‐catenin. Moreover, the augmented protein levels of cleaved caspase‐3 and Bax on account of sh‐DLGAP1‐AS1 could be partly reversed by pcDNA3.1‐ROCK1 or pcDNA3.1‐NFE2L1, but totally recovered by cotransfection of pcDNA3.1‐ROCK1 and pcDNA3.1‐NFE2L1. In addition, the repressed Bcl2 and C‐myc level caused by DLGAP1‐AS1 knockdown was totally reversed by overexpression of ROCK1 and NFE2L1 (Figure 5d). Collectively, DLGAP1‐AS1 activated Wnt signaling pathway via regulating NFE2L1 and ROCK1.

### 3.6 DLGAP1‐AS1 accelerates GBM cell proliferation and inhibits cell apoptosis by upregulating NFE2L1 and ROCK1

**FIGURE 6:**
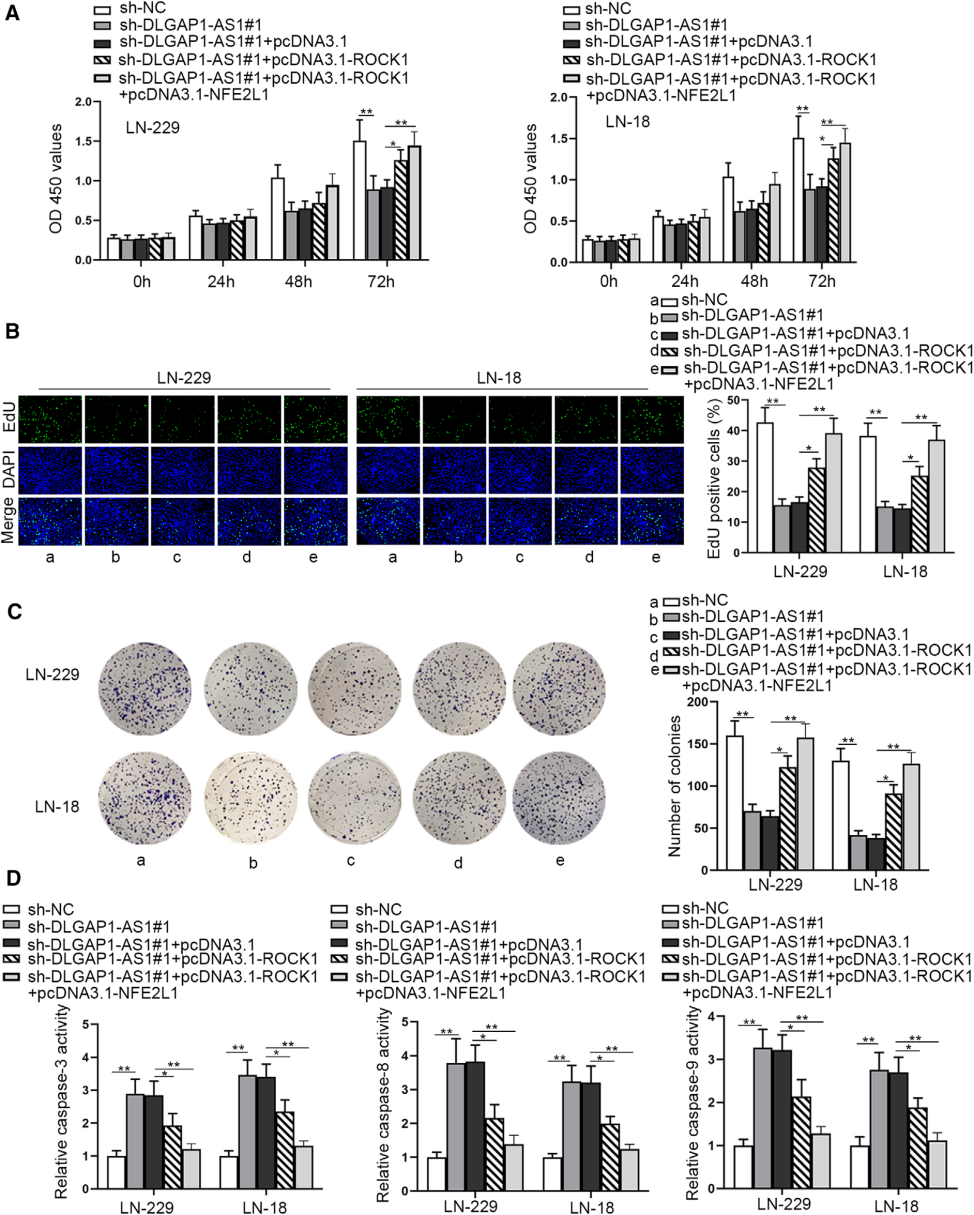
DLGAP1‐AS1 accelerates GBM cell proliferation and inhibits cell apoptosis by upregulating NFE2L1 and ROCK1. (a) CCK‐8 assays were applied for measuring cell viability in different groups. (b–c) EdU and colony formation assays were done for the investigation into cell proliferation on account of the transfection of different plasmids. (d) Caspase‐3/8/9 activity assay was employed for detecting cell apoptosis under different transfection conditions. ^*^ *p* < .05, ^**^ *p* < .01

In the end, we implemented the rescue assays to validate the ceRNA network of DLGAP1‐AS1 in GBM cells. It was indicated from CCK‐8 assays that cell viability could be repressed as a result of sh‐DLGAP1‐AS1 transfection, which was partly recovered by pcDNA3.1‐ROCK1, but completely recovered by cotransfection of pcDNA3.1‐ROCK1 and pcDNA3.1‐NFE2L1 (Figure 6a). Following, EdU assay and colony formation assay proved that sh‐DLGAP1‐AS1‐inhibited cell proliferation could be partly recovered by overexpression of ROCK1, while it could be totally recovered by overexpression of both ROCK1 and NFE2L1 (Figure 6b‐c). Finally, caspase‐3/8/9 assays and flow cytometry assay indicated that upregulation of ROCK1 and NFE2L1 could totally counteract the promoting role of silencing DLGAP1‐AS1 on cell apoptosis, which could only be partly reversed by upregulation of ROCK1 (Figures 6d and S2d). In addition, we performed further rescue assays by cotransfecting sh‐DLGAP1‐AS1 to pcDNA3.1‐ROCK1/pcDNA3.1‐NFE2L1 transfected cells. As a result, CCK‐8 assay indicated cell viability enhanced by pcDNA3.1‐ROCK1/pcDNA3.1‐NFE2L1 was reduced by sh‐DLGAP1‐AS1 (Figure S3a). EdU assay and colony formation assay proved DLGAP1‐AS1 knockdown could reverse the promoted cell proliferation induced by ROCK1/NFE2L1 augment (Figure S3b‐c). Caspase‐3/8/9 assays and flow cytometry assay verified cell apoptosis reduced by transfection of pcDNA3.1‐ROCK1/pcDNA3.1‐NFE2L1 could be counteracted by sh‐DLGAP1‐AS1 (Figure S3d‐e). Also, we overexpressed DLGAP1‐AS1 to rescue sh‐ROCK1/sh‐NFE2L1 transfected cells. ROCK1/NFE2L1 inhibition efficiency was firstly determined, and sh‐ROCK1#1/sh‐NFE2L1#1 was selected for subsequent rescue experiments for the higher efficiency (Figure S4a). The result of CCK‐8 assay showed decreased cell viability caused by ROCK1/NFE2L1 knockdown could be rescued by DLGAP1‐AS1 overexpression (Figure S4b). EdU assay and colony formation assay certified cell proliferation hampered by ROCK1/NFE2L1 depletion was reversed by DLGAP1‐AS1 overexpression (Figure S4c‐d). Cell apoptosis enhanced by depletion of ROCK1/NFE2L1 was also reversed by cotransfection of pcDNA3.1‐DLGAP1‐AS1 (Figure S4e‐f). Taken together, DLGAP1‐AS1 accelerated GBM cell proliferation and curbed cell apoptosis by upregulating NFE2L1 and ROCK1.

## 4 DISCUSSION

GBM is the primary malignant tumor in the central nervous system with high aggressiveness and mortality. The current treatment methods can not effectively improve the survival rate of GBM patients. Therefore, exploring new treatment strategies can help to improve the current predicament. With the biological function of lncRNAs being gradually discovered, more and more researches begin to focus on lncRNAs in tumorigenesis. Even some lncRNAs have been identified as biomarkers for the diagnosis, treatment and prognosis of assorted cancer. For example, circulating lncRNA XLOC_009167 served as a diagnostic biomarker to predict lung cancer (Jiang et al., 2018). LncRNA HOTAIR was confirmed as prognostic circulating marker and potential therapeutic target in tumor patients (Botti et al., 2017). In our research, we investigated DLGAP1‐AS1 in GBM cells. In accordance with experimental results, we discovered that DLGAP1‐AS1 was notably upregulated in GBM tissues and cells. Importantly, DLGAP1‐AS1 inhibition could repress cell proliferation and accelerate cell apoptosis. In previous studies, DLGAP1‐AS1 was conformed to accelerate tumorigenesis and EMT process in hepatocellular carcinoma (Lin et al., 2020) and facilitate the cell invasion in gastric cancer (Deng et al., 2020). These researches are in consistence with our findings that DLGAP1‐AS1 is highly expressed in GBM cells and exerts the carcinogenic effect.

Next, our research proved that DLGAP1‐AS1 could interact with miR‐515‐5p in GBM cells by acting as a ceRNA (Paraskevopoulou & Hatzigeorgiou, 2016). In recent years, ceRNA network is widely studied in human cancer cells and it can be involved in the regulation of cancer progression (Qi et al., 2015). Importantly, lncRNA can function as a ceRNA to sponge miRNAs, so as to release the inhibition on mRNA expression which is caused by miRNA at posttranscriptional level (Sen et al., 2014). The key of posttranscriptional regulation lies in the subcellular localization of lncRNA. If lncRNA is distributed in the cytoplasm, it can posttranscriptionally regulate gene expression. After we determined the subcellular localization of DLGAP1‐AS1 in GBM cells, we evaluated possibility of DLGAP1‐AS1 to function as a ceRNA. AGO2‐RIP assay proved DLGAP1‐AS1 could exist in RISC complex and might bind with the specific miRNA. Through utilizing bioinformatics tools and performing mechanism assays, we confirmed that DLGAP1‐AS1 could bind to miR‐515‐5p in GBM cells by acting as a ceRNA. Besides, miR‐515‐5p was found to be lowly expressed in GBM cells and its overexpression could repress cell proliferation and promote cell apoptosis. Previous reports have shown that miR‐515‐5p plays an anticancer role in some cancers. For example, miR‐515‐5p was confirmed as a tumor suppressor in prostate cancer (Zhang et al., 2019). Also, miR‐515‐5p could suppress cell migration of breast cancer (Pardo et al., 2016).

ROCK1 is a famous oncogene and its role has been identified in assorted human cancers. In nonsmall‑cell lung cancer cells, ROCK1 was discovered to be expressed at high level and promote cell migration and invasion by the PTEN/PI3K/FAK pathway (Hu et al., 2019). It was also reported that ROCK1 could be targeted by miR‐145 and accelerate cell growth of breast cancer (Zheng et al., 2016). Importantly, ROCK1 was reported to facilitate cell migration and invasion in GBM (An et al., 2013). Similarly, we proved that ROCK1 was highly expressed in GBM cells, and it could be targeted by miR‐515‐5p. On the other hand, we also proved that NFE2L1 was another target gene of miR‐515‐5p in GBM cells. Besides, both of ROCK1 and NFE2L1 were confirmed to be positively associated with DLGAP1‐AS1 and negatively associated with miR‐515‐5p. It was reported that NFE2L1was involved in SLCO4A1‐AS1‐mediated activation of WNT pathway and accelerated lung adenocarcinoma progression. In like manner, ROCK1 was confirmed as the main genes of the Wnt signaling pathway (Mohammadi‐Yeganeh et al., 2019; Xie et al., 2020). Thus, we evaluated whether DLGAP1‐AS1 could activate Wnt signaling pathway through regulating ROCK1 and NFE2L1. Through TOP/FOP assay and western blot, we proved that DLGAP1‐AS1 could activate Wnt signaling pathway through upregulating ROCK1 and NFE2L1. Wnt signaling pathway is one of the most crucial signaling pathways and its activation is also frequent in the progression of assorted cancers (Taciak et al., 2018). Anomalous activation of Wnt pathway results in the accumulation of β‐catenin in the nucleus and accelerates the transcription of numerous oncogenes like C‐myc (Shang et al., 2017). In our study, we detected that the protein level of nuclear β‐catenin was reduced by sh‐DLGAP1‐AS1, and then partially reversed by ROCK1 overexpression or NFE2L1 overexpression. However, it could be totally reversed when ROCK1 and NFE2L1 were overexpressed at the same time. In the final rescue assays, we also found that cell proliferation inhibited by DLGAP1‐AS1 depletion could be totally recovered by upregulation of ROCK1 and NFE2L1 at the same time. Meanwhile, rescue assay also manifested that DLGAP1‐AS1 knockdown or overexpression could also affect the functions of ROCK1 and NFE2L1 in GBM cells.

Taken together, our research proved the upregulation of DLGAP1‐AS1 in GBM cells. Meanwhile, DLGAP1‐AS1 could act as a ceRNA to sponge miR‐515‐5p and regulate ROCK1 and NFE2L1. Furthermore, DLGAP1‐AS1 could accelerate GBM cell proliferation through upregulating ROCK1 and NFE2L1 expression and activating Wnt signaling pathway. But there was lack of clinical samples in this study, which should be involved in future research. Nevertheless, our discoveries could still provide a novel prospective for developing therapeutic targets for GBM.

## FUNDING

This study was supported by Efficacy evaluation of precision therapy for children brain stem glioma (DIPG).

5

### PEER REVIEW

The peer review history for this article is available at https://publons.com/publon/10.1002/brb3.2321

## Supporting information

Supporting information
**Figure S1** (a) DLGAP1‐AS1 (ENST00000576606) expression in GBM tissues was obtained from GEPIA. (b) DLGAP1‐AS1 (ENST00000576606) expression in GBM cell lines and NHAs was detected via RT‐qPCR

Supporting information
**Figure S2** (a) Flow cytometry analysis was utilized to detect apoptosis of GBM cells after DLGAP1‐AS1 silence. (b) The overall survival of GBM patients with low or high DLGAP1‐AS1 level was obtained from GEPIA. (c) Cell apoptosis was detected via flow cytometry analysis after overexpressing miR‐515‐5p. (d) Cell apoptosis was examined by flow cytometry analysis in different groups. ^*^ *p* < .05, ^**^ *p* < .01

Supporting information.
**Figure S3** (a) CCK‐8 assays were applied for measuring cell viability in different groups. (b–c) EdU and colony formation assays were utilized to estimate cell proliferation in different groups. (d) Caspase‐3/8/9 activity assay was employed for detecting cell apoptosis in different groups. (e) Flow cytometry assay was conducted to detect cell apoptosis. ^**^ *p* < .01

Supporting information
**Figure S4** (a) ROCK1/NFE2L1 inhibition efficiency was determined via RT‐qPCR analysis. (b) CCK‐8 assays were applied for measuring cell viability in different groups. (c–d) EdU and colony formation assays were utilized to estimate cell proliferation in different groups. (e) Caspase‐3/8/9 activity assay was employed for detecting cell apoptosis in different groups. (f) Flow cytometry assay was conducted to detect cell apoptosis. ^**^ *p* < .01
