# Supplementary figures and images for: LncRNA DLGAP1‐AS1 accelerates glioblastoma cell proliferation through targeting miR‐515‐5p/ROCK1/NFE2L1 axis and activating Wnt signaling pathway
Source: Brain Behav. 2021 Sep 18;11(10):e2321. doi: 10.1002/brb3.2321 (PMC8553332; doi:10.1002/brb3.2321)

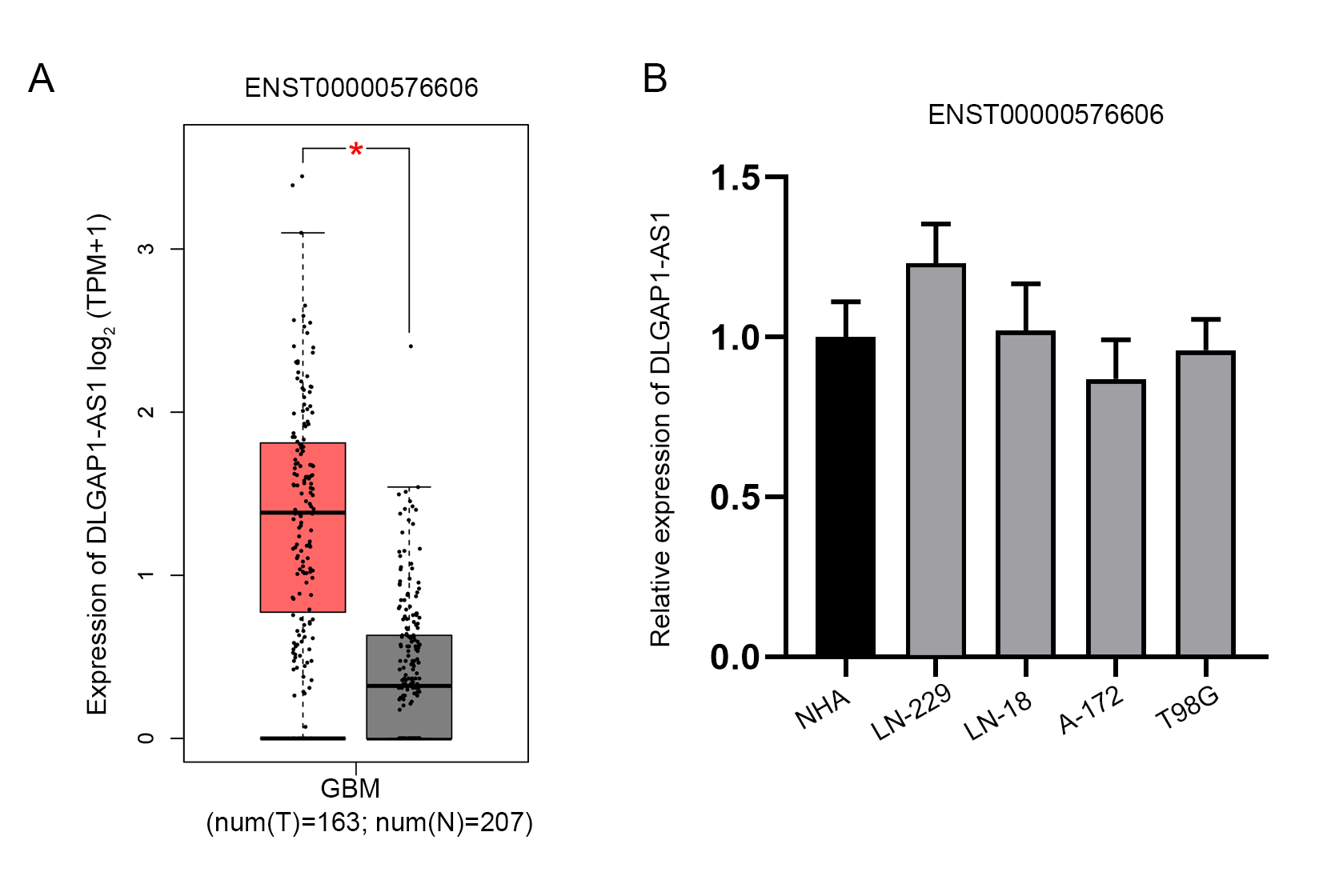

Supplement: Supplementary file 1 — Supporting information Figure S1 (a) DLGAP1‐AS1 (ENST00000576606) expression in GBM tissues was obtained from GEPIA. (b) DLGAP1‐AS1 (ENST00000576606) expression in GBM cell lines and NHAs was detected via RT‐qPCR [file BRB3-11-e2321-s001.tif]

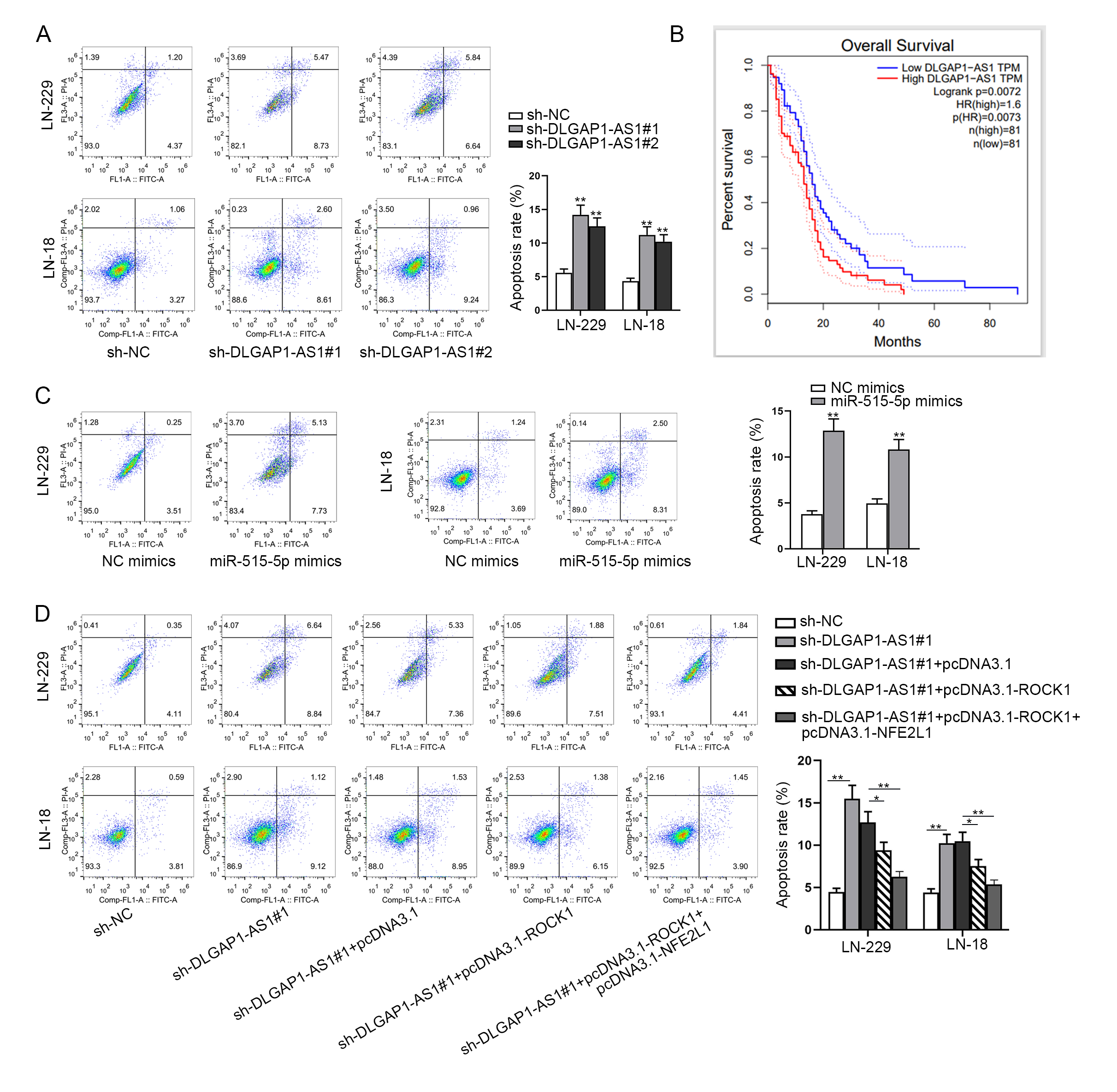

Supplement: Supplementary file 2 — Supporting information Figure S2 (a) Flow cytometry analysis was utilized to detect apoptosis of GBM cells after DLGAP1‐AS1 silence. (b) The overall survival of GBM patients with low or high DLGAP1‐AS1 level was obtained from GEPIA. (c) Cell apoptosis was detected via flow cytometry analysis after overexpressing miR‐515‐5p. (d) Cell apoptosis was examined by flow cytometry analysis in different groups. * p < .05, ** p < .01 [file BRB3-11-e2321-s002.tif]

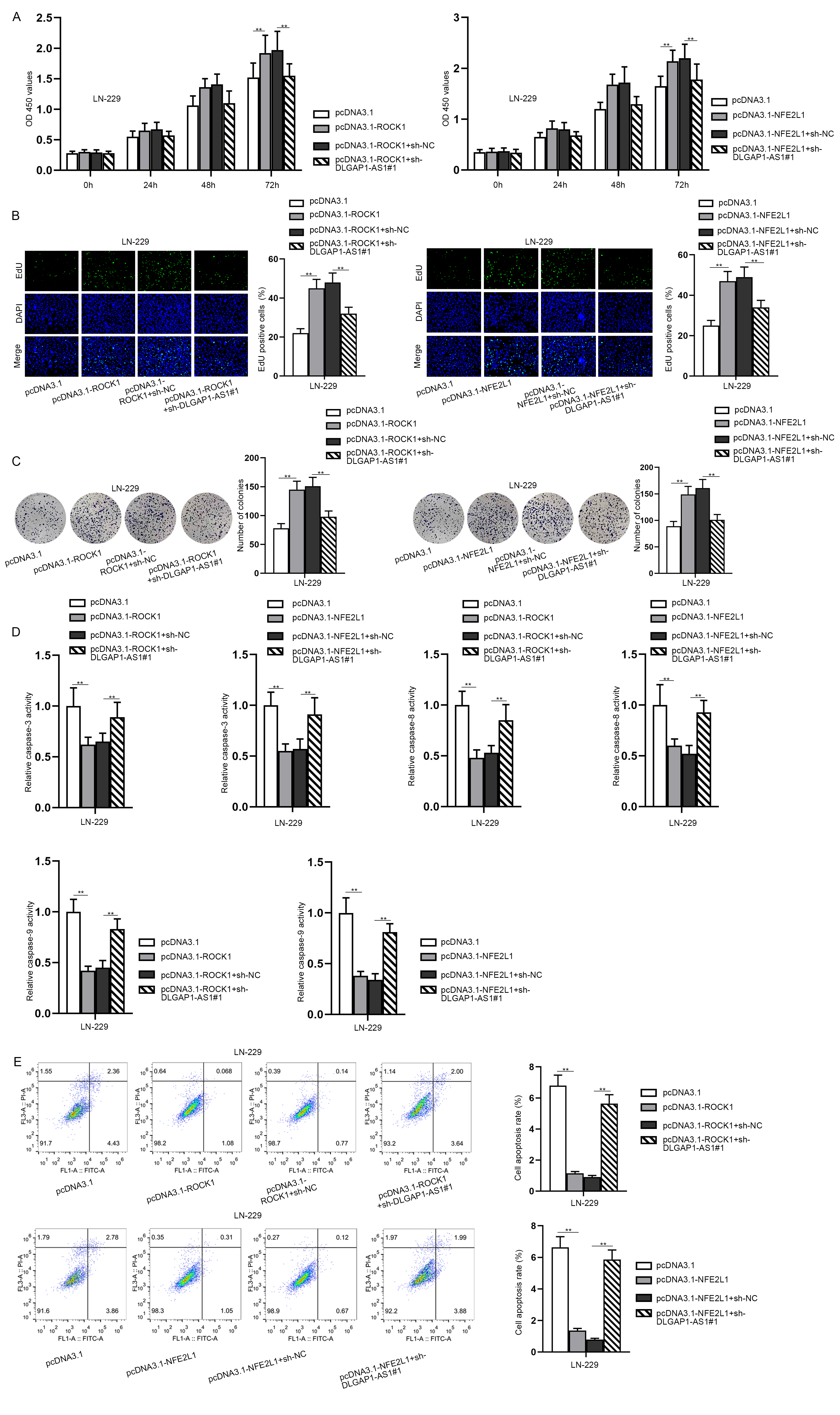

Supplement: Supplementary file 3 — Supporting information. Figure S3 (a) CCK‐8 assays were applied for measuring cell viability in different groups. (b–c) EdU and colony formation assays were utilized to estimate cell proliferation in different groups. (d) Caspase‐3/8/9 activity assay was employed for detecting cell apoptosis in different groups. (e) Flow cytometry assay was conducted to detect cell apoptosis. ** p < .01 [file BRB3-11-e2321-s004.tif]

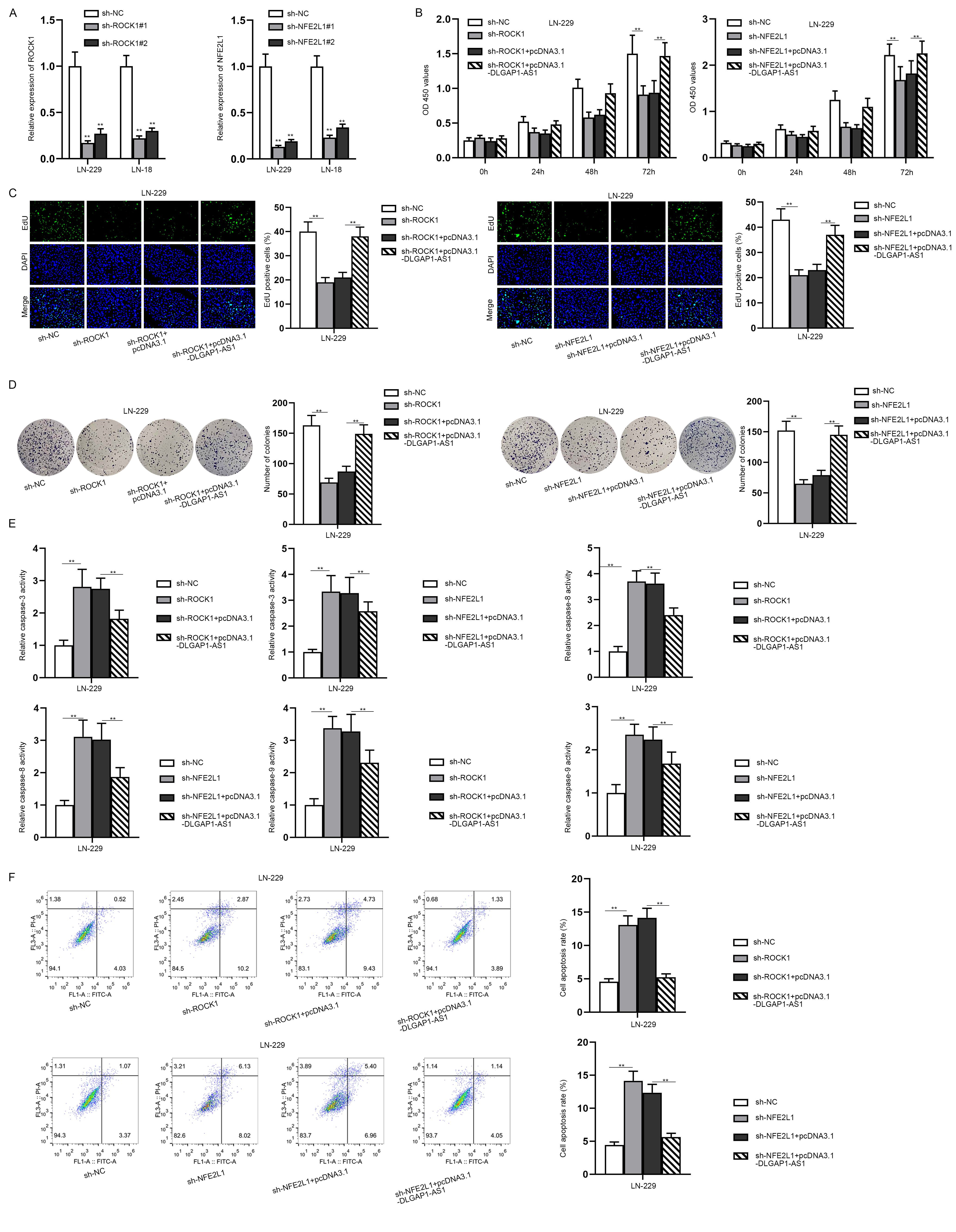

Supplement: Supplementary file 4 — Supporting information Figure S4 (a) ROCK1/NFE2L1 inhibition efficiency was determined via RT‐qPCR analysis. (b) CCK‐8 assays were applied for measuring cell viability in different groups. (c–d) EdU and colony formation assays were utilized to estimate cell proliferation in different groups. (e) Caspase‐3/8/9 activity assay was employed for detecting cell apoptosis in different groups. (f) Flow cytometry assay was conducted to detect cell apoptosis. ** p < .01 [file BRB3-11-e2321-s003.tif]
